# Macrophage inhibits the osteogenesis of fibroblasts in ultrahigh molecular weight polyethylene (UHMWPE) wear particle-induced osteolysis

**DOI:** 10.1186/s13018-019-1119-8

**Published:** 2019-03-18

**Authors:** Pengfei Lei, Zixun Dai, Yu Shrike Zhang, Hua Liu, Wanting Niu, Kun Li, Long Wang, Yihe Hu, Jie Xie

**Affiliations:** 1 Department of Orthopedics, Xiangya Hospital of Central South University, 87 Xiangya Road, Changsha, 410008 Hunan People’s Republic of China; 2 Department of Orthopedics, Hunan Cancer Hospital and The Affiliated Cancer Hospital of Xiangya School of Medicine of Central South University, Changsha, 410008 Hunan People’s Republic of China; 3 Centre for Biomaterials Innovation, Department of Medicine, Brigham and Women’s Hospital, Harvard Medical School, Cambridge, MA 02139 USA; 4 Dr. Li Dak Sum & Yip Yio Chin Center for Stem Cell and Regenerative Medicine, School of Medicine, Zhejiang University, Hangzhou, 310058 People’s Republic of China; 5 Key Laboratory of Tissue Engineering and Regenerative Medicine of Zhejiang Province, Hangzhou, 310058 People’s Republic of China; 6 Harvard-MIT Division of Health Sciences and Technology, Tissue Engineering Lab, Cambridge, USA; 7 VA Boston Healthcare System, West Roxbury, MA 02132 USA; 8 Department of Orthopedics, Brigham and Women’s Hospital, Harvard Medical School, Cambridge, MA 02139 USA

**Keywords:** Ultrahigh molecular weight polyethylene, Wear particles, Fibroblast, Macrophage, Osteogenesis, Osteolysis

## Abstract

**Background:**

In the ultrahigh molecular weight polyethylene (UHMWPE) prosthetic environment, fibroblasts affected by wear particles have the capacity of osteogenesis to reduce osteolysis. We aimed to assess the effects of macrophages on the osteogenic capability of fibroblasts treated with UHMWPE wear particles.

**Methods:**

The effect of different concentrations of UHMWPE (0, 0.01, 0.1, and 1 mg/ml, respectively) on macrophage proliferation were validated by MTT assay to determine the optimum one. The fibroblasts viability was further determined in the co-culture system of UHMWPE particles and macrophage supernatants. The experiment was designed as seven groups: (A) fibroblasts only; (B) fibroblasts + 1 mg/ml UHMWPE particles; and (C1–C5) fibroblasts + 1/16, 1/8, 1/4, 1/2, and 1/1 supernatants of macrophage cultures stimulated by 1 mg/ml UHMWPE particles vs. fibroblast complete media, respectively. Alizarin red staining was used to detect calcium accumulation. The expression levels of osteogenic proteins were detected by Western blot and ELISA, including alkaline phosphatase (ALP) and osteocalcin (OCN).

**Results:**

The concentration of 0.1 mg/ml was considered as the optimum concentration for macrophage proliferation due to the survival rate and was highest among the four concentrations. Fibroblast viability was better in the group of fibroblasts + 1/16 ratio of macrophage supernatants stimulated by 1 mg/ml of UHMWPE particles than the other groups (1:8, 1:4, 1:2, 1:1). ALP and OCN expressions were significantly decreased in the group of fibroblasts + 1/4, 1/2, and 1/1 supernatants stimulated by 1 mg/ml of UHMWPE particles compared with other groups (1/8, 1/16) and the group of fibroblasts + 1 mg/ml UHMWPE (*p* < 0.5).

**Conclusions:**

Macrophages are potentially involved in the periprosthetic osteolysis by reducing the osteogenic capability of fibroblasts treated with wear particles generated from UHMWPE materials in total hip arthroplasty.

## Introduction

Periprosthetic osteolysis provoking aseptic loosening of the implant results in the failure of total hip arthroplasty (THA) and boosts a requirement of revision surgery [1]. Ultrahigh molecular weight polyethylene (UHMWPE) has been widely used as prosthesis materials for total joint replacements due to its excellent biocompatibility, low friction coefficient, and corrosion resistance properties [2]. Wear-generated particle debris promotes periprosthetic osteolysis, which is referred to as the progressive insidious bone resorption [3]. The prosthesis wear particles in the interfacial membrane (bone/prosthesis interface) stimulate various cell types in the periprosthetic area, such as macrophages and fibroblasts, leading to osteolysis [1]. Previous studies reported that interface membrane fibroblasts, affected by inflammatory cytokines, especially by wear particles, significantly contribute to osteoclast genesis and periprosthetic osteolysis [4, 5]. Many researchers have found that macrophages play a critical role in periprosthetic osteolysis by actively phagocytosing wear particles and produce inflammatory cytokines, leading to the imbalance of osteoclastic bone resorption and osteoblastic bone formation [6–8].

Macrophage is one of the major cell components in the prosthetic milieu and plays a critical role in the different foreign body responses. During the process of phagocytosis, macrophages released prostaglandins, cytokines, metalloproteinases, and lysosomal enzymes by wear particle stimulation which leads to activation of bone resorbing pathways [9, 10]. Macrophages were also reported to enhance osteogenesis of mouse clonal osteoblast-like cells (MC3T3) in vitro in the presence of calcium silicate cement via the bone morphogenetic protein-2 (BMP2) signaling pathway [11, 12]. So far, most studies focus on the effect of UHMWPE particles on macrophage secretions [13, 14]. The effects of macrophages on fibroblasts induced by UHMWPE particles remain unclear. As previously reported, the secretion of macrophages plays an important role in enhancing osteogenesis of fibroblasts [12]. Our previous results demonstrated that fibroblasts derived from the synovial membrane can be converted into osteoblasts by UHMWPE wear particles [15]. We proposed a hypothesis that macrophages participate in the periprosthetic osteolysis process by reducing the osteogenic capability of fibroblasts treated with UHMWPE wear particles. In the present study, we investigate the role of macrophage secretion on the in vitro biological activities of fibroblasts, especially the osteogenic capability, upon the stimulation by the UHMWPE wear particles.

## Materials and methods

### Fibroblasts and macrophage cultures

Fibroblasts were isolated from the hip synovial membrane of patients with femoral neck fracture during THA. Fibroblasts were cultured in Dulbecco’s modified Eagle’s medium (DMEM) containing 100 U/mL penicillin and 100 μg/mL streptomycin supplemented with 20% heat-inactivated fetal bovine serum (FBS) (Gibco, USA). Murine macrophage cell line Ana-1 (GNM2, Cell bank, China) were cultured in RPMI-1640 (Gibco, NY, Grand Island, USA) supplemented with 1.5 g/L NaHCO3, 2.5 g/L glucose, 0.11 g/L sodium pyruvate, and 10% FBS at 37 °C, 5% CO_2_.

### Preparation of UHMWPE particles

UHMWPE particles with an average diameter of 6.54 μm ± 4.43 μm were prepared using a ball milling technique and imaged by scanning electron microscope (SEM, Quanta-200, FEI, Netherlands). UHMWPE wear particles in diameters of 53–75 μm were purchased from Sigma-Aldrich, USA. UHMWPE particles could mimic UHMWPE lining separated during the artificial hip revision procedures, which were analyzed and identified by using Raman spectrometer (HORIBA JobinYvon S.A.S, France). All the procedures were performed as previously reported [15]. Informed consent was obtained from all patients. The study was approved by the Ethics Committee of XiangYa Hospital, Central South University, China. The procedures were performed in accordance with the international ethics standards of human trials.

### 3-(4,5-Dimethylthiazol-2-yl)-2,5-diphenyltetrazolium bromide (MTT) assay

To determine the proliferation of macrophages in different concentrations of UHMWPE particles and to explore the optimum concentration, MTT assay was adopted to detect the cell viability [16]. Macrophages of logarithmic growth phase were seeded in a 96-well plate at a density of 1 × 10^4^ cells/100 μl per well. Various concentrations of UHMWPE particles (0, 0.01, 0.1, and 1 mg/ml) were incubated with macrophages for 24 h at 37 °C in a humidified incubator at the atmosphere of 5% CO_2_. Macrophages without UHMWPE particles were treated as the negative control. Serum-free medium (100 μl) and 5 mg/ml MTT (20 μl, Sigma, USA) was further added into each well and subsequently incubated for 4 h. Following the removal of supernatant, the insoluble formazan crystals were dissolved in 150 μl dimethyl sulfoxide (DMSO), and absorbance at days 1, 3, 5, and 7 was measured by microplate reader (Bio-Rad, USA) at the 570-nm wavelength. Cell survival rate is (%) = [OD (particle-treated group)/OD (control)] × 100%.

To investigate the effect of macrophages treated by 1 mg/ml UHMWPE particles on the viability of fibroblasts, the supernatant was collected at 48 h. According to the different proportions of macrophage supernatants vs. fibroblast media, the co-culture system was divided into seven groups as follows: (A) fibroblasts only, (B) fibroblasts + 1 mg/ml UHMWPE particles, and (C1–C5) fibroblasts + 1/16, 1/8, 1/4, 1/2, and 1/1 of macrophage supernatants stimulated by 1 mg/ml of UHMWPE particles, respectively. The ratios of 1/16, 1/8, 1/4, 1/2, and 1/1 were defined as the proportion of macrophage supernatants vs. fibroblast complete media. MTT assays were performed as previously mentioned.

### Alizarin red staining

To explore the effect of macrophage secretion on fibroblast osteogenic differentiation, alizarin red staining was used to detect the matrix mineralization, a process that occurs at the later stages of bone formation in all groups. In brief, cell specimens were washed with PBS and were fixed in 95% ethanol for 10 min. The sections were stained by alizarin red solution [17].

### Western blot

Extracted protein was harvested at day 5 from the following co-culture systems: (A) fibroblasts only, (B) fibroblasts + 1 mg/ml UHMWPE particles, and (C1) fibroblasts + 1/16 macrophage supernatants stimulated by 1 mg/ml of UHMWPE particles. The protein was extracted with RIPA lysis buffer with phenylmethylsulfonyl fluoride (PMSF). The total protein was quantified by using a BCA Protein Assay kit (Thermo Scientific, USA) according to the manufacturer’s instructions [18]. Thereafter, 50 μg of extracted protein was boiled at 99 °C for 5 min in 1 × loading buffer and chilled down on ice. The electrophoresis was performed in 10% SDS-PAGE gels and then transferred onto polyvinylidene difluoride (PVDF) membranes. Non-specific binding was blocked by incubating with 5% fat-free milk in TBST buffer. After blocking the nonspecific sites with a blocking solution, the primary antibodies were incubated overnight at 4 °C, including anti-alkaline phosphatase (ALP) (1:10000, Abcam, ab133602, USA), anti-osteocalcin (OCN) (1:400; Santa Cruz, sc-74495, USA), and anti-GAPDH (1:800; Santa Cruz, sc-365062, USA). Subsequently, the membranes were washed with PBST followed by incubation with secondary goat anti-rabbit or goat anti-mouse horseradish peroxidase (HRP)-conjugated antibodies (Sigma, USA) for 1 h at room temperature. The blots were detected by an enhanced chemiluminescence (ECL) reagent (Pierce, USA). Bands were quantified by using the software of Gel-pro Analyzer 4.0.

### Enzyme-linked immunosorbent assay (ELISA)

Serum-free DMEM was used for determining the OCN and ALP in the following co-culture systems: (A) fibroblasts only, (B) fibroblasts + 1 mg/ml UHMWPE particles, and (C1) fibroblasts + 1/16 supernatants stimulated by 1 mg/ml UHMWPE particles. OCN were assessed by enzyme-linked immunosorbent assay kit (SEA471Hu, Cloud-Clone Corp, USA). ALP activity was quantified by ELISA kit (SEB472Hu, Cloud-Clone Corp, USA) according to the instructions of the manufacturers. The optical densities (OD) were determined using a plate reader at 450 nm as previously reported [11, 19].

### Statistical analysis

All quantitative data were expressed as the mean ± standard deviation (SD). Statistical analysis was performed with SPSS 17.0 software. Mean comparison was detected using one-way analysis of variance (ANOVA, *n* = 3), and *p* < 0.05 was considered statistically significant.

## Results

### A higher survival rate of macrophage at lower concentration of UHMWPE particles

**Fig. 1:**
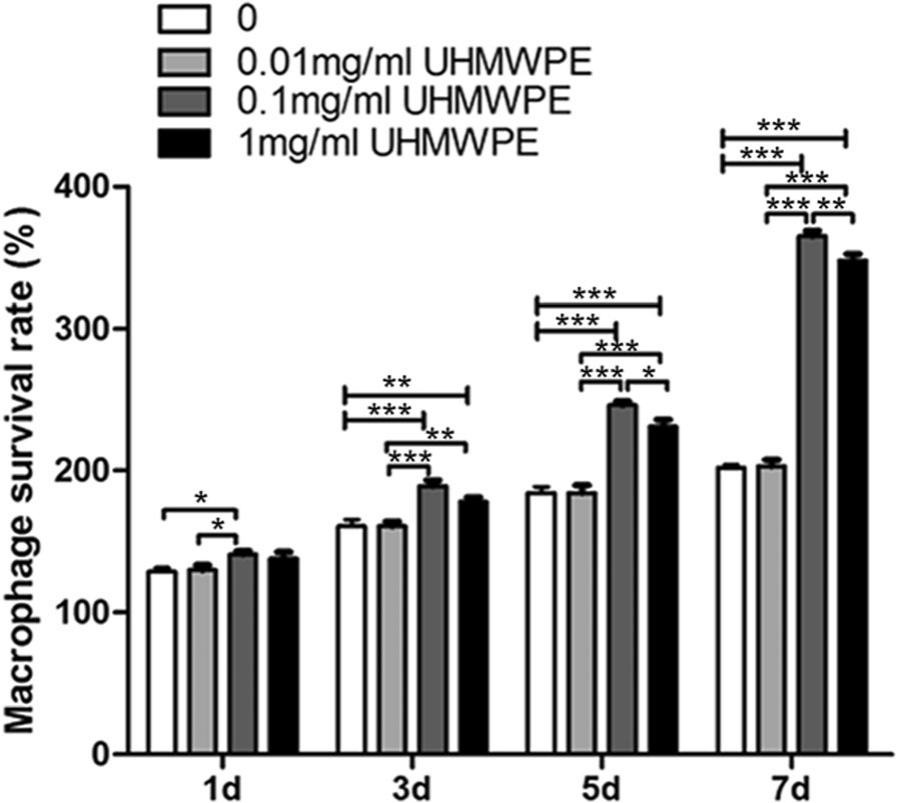
Viability of macrophages treated with different concentrations of UHMWPE particles at days 1, 3, 5, and 7. One asterisk indicates *p* < 0.05, two asterisks indicate *p* < 0.01, and three asterisks indicate *p* < 0.001 between two groups

Cell viability of macrophages was determined in the different concentrations of UHMWPE particles (Fig. 1). There was no statistical difference in the survival rate of macrophages between treated with 0.01 mg/ml UHMWPE particles and UHMWPE free controls at days 1, 3, 5, 7. However, there were significant differences in both macrophages + 0.1 mg/ml UHMWPE particles group and macrophages + 1 mg/ml UHMWPE particles group compared to macrophages only group (*p* < 0.01). In addition, the survival rate of macrophages was higher in macrophages + 0.1 mg/ml UHMWPE particles group than that in macrophages + 1 mg/ml UHMWPE particles group (*p* < 0.05). The results revealed that 0.1 mg/ml UHMWPE particles was the optimum concentration that stimulated for macrophage proliferation (Fig. 1).

### Macrophage supernatant in the existence of UHMWPE particles reduced fibroblast survival rate

**Fig. 2:**
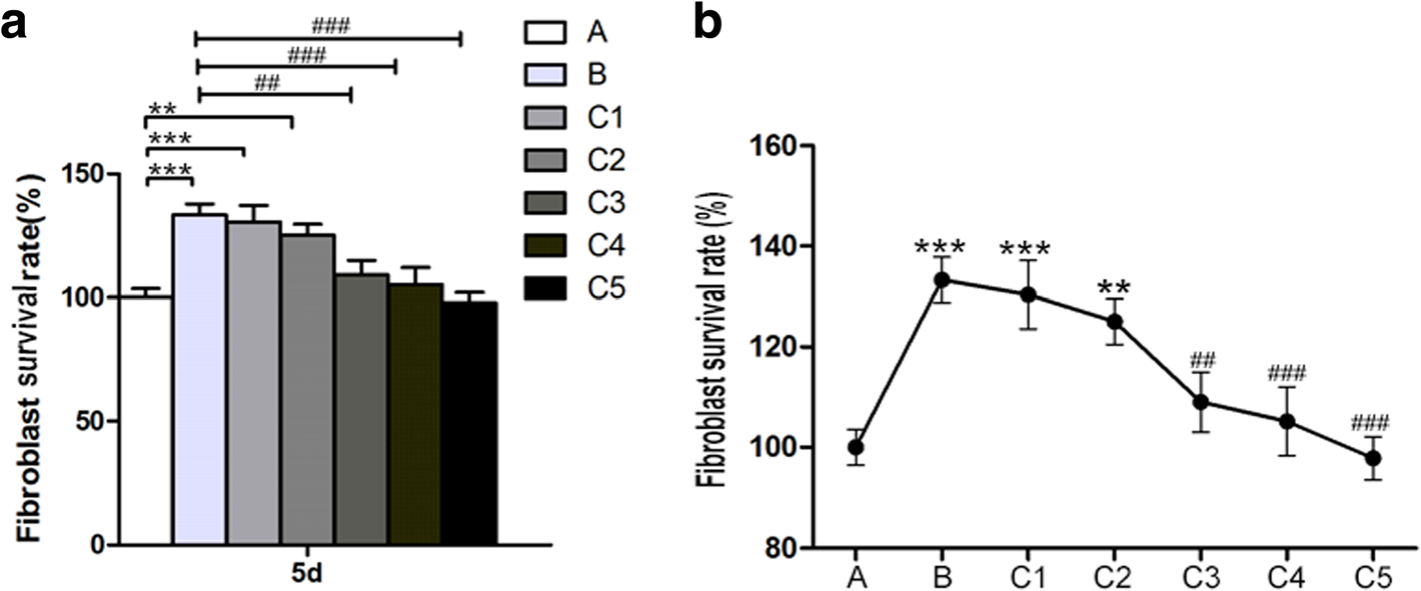
Macrophage supernatants’ effect on the viability of fibroblasts treated with 1 mg/ml UHMWPE particles at day 5. Two asterisks indicate *p* < 0.01, three asterisks indicate *p* < 0.001 versus A (fibroblasts only); Two number signs indicate *p* < 0.01, three number signs indicate *p* < 0.001 versus B (fibroblasts + 1 mg/ml UHMWPE particles). **a**. fibroblasts only; **b**. fibroblasts + 1 mg/ml UHMWPE particles; C1-C5. fibroblasts + 1/16, 1/8, 1/4, 1/2, 1/1 of macrophage supernatants stimulated by 1mg/ml of UHMWPE particles, respectively

It is obvious that the survival rate of fibroblast was significantly higher in fibroblasts + 1 mg/ml UHMWPE particles group than that in fibroblasts with only control group (*p* < 0.001, group A vs. B). Cell viability of fibroblasts treated with 1 mg/ml UHMWPE particles was reduced as supernatants from macrophage cultures treated with 1 mg/ml of UHMWPE particles were added to the culture media (*p* < 0.01, Fig. 2a). Fibroblast survival rate was gradually decreased as the ratio of the macrophage supernatants increased. The results showed that proliferated or survived fibroblasts were better in the group of 1/16 macrophage supernatants from co-culture with 1 mg/ml of UHMWPE particles compared to other macrophage supernatant proportions accounting for fibroblasts’ complete media (*p* < 0.01, Fig. 2b).

### Macrophage supernatant in the existence of UHMWPE wear particles reduced osteogenic capacity of fibroblasts

**Fig. 3:**
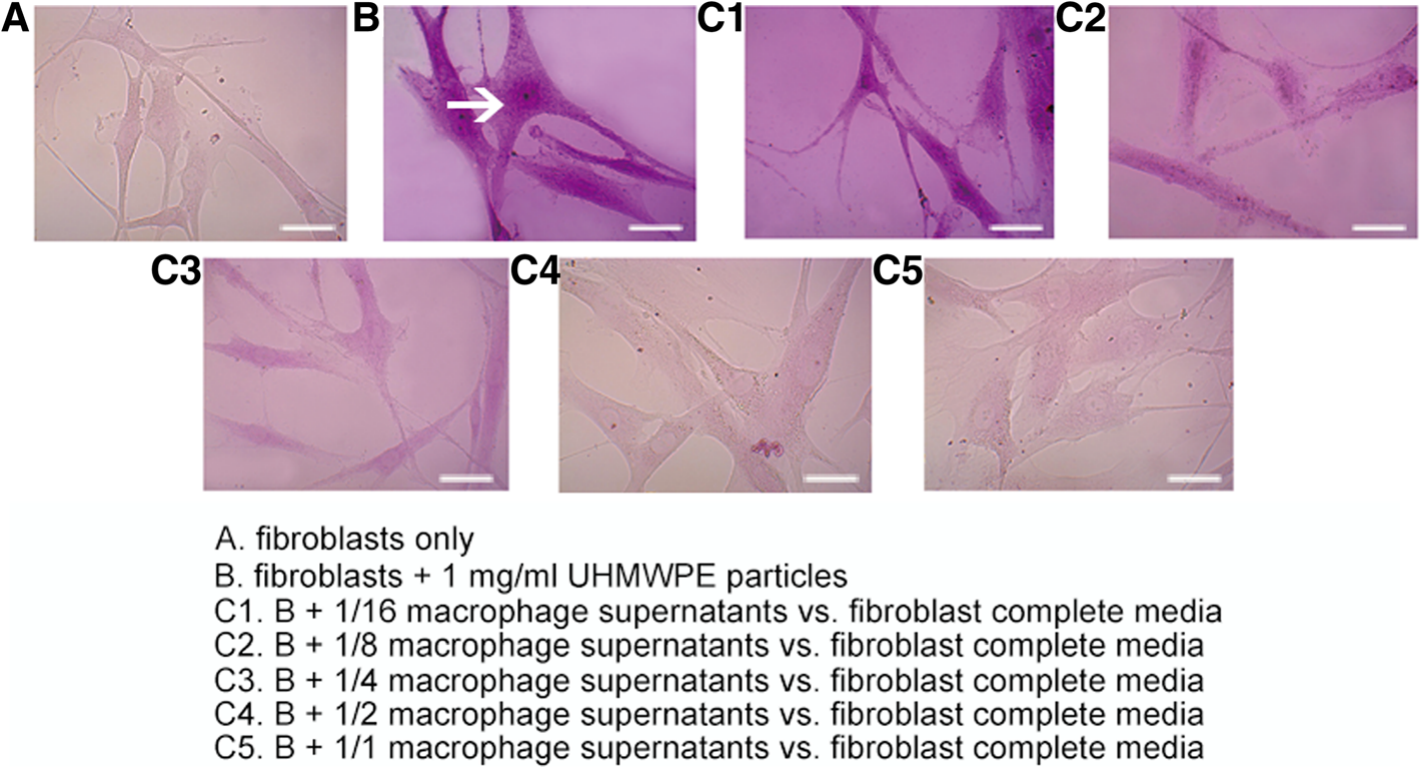
Alizarin red staining of fibroblast treated with UHMWPE particles and added with macrophage supernatants (× 40)

Calcium accumulation (red aggregate deposition) was more pronounced in the fibroblasts + 1 mg/ml UHMWPE particles group compared to fibroblasts with only control group. However, the staining intensity gradually decreased as the ratio of macrophage supernatants increased (Fig. 3).

### Macrophage supernatant upon the existence of UHMWPE wear particles reduced osteogenic protein secretion of fibroblasts

**Fig. 4:**
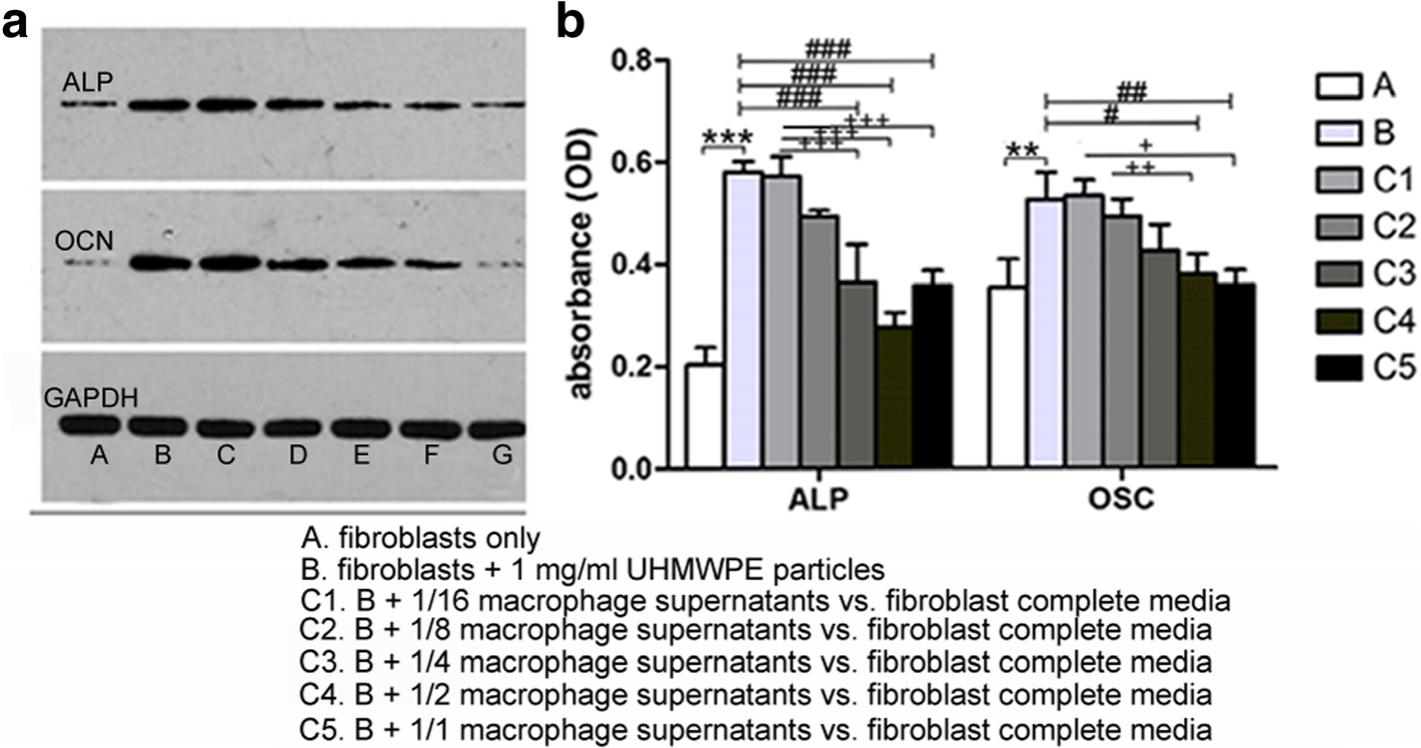
Osteogenic protein level of fibroblasts treated with UHMWPE particles and added with macrophage supernatants. **a** Western blot, **b** ELISA. One asterisk indicates *p* < 0.05, two asterisks indicate *p* < 0.01, three asterisks indicate *p* < 0.001 versus A (fibroblasts only); one number sign indicates *p* < 0.05, two number signs indicate *p* < 0.01, three number sign indicate *p* < 0.001 versus B (fibroblasts + 1 mg/ml UHMWPE particles). One plus sign indicates *p* < 0.05, two plus signs indicate *p* < 0.01, ^t^hree plus signs indicate *p* < 0.001 versus C1 (fibroblasts + 1 mg/ml UHMWPE particles + 1/16 macrophage supernatants vs. fibroblast media)

Protein expressions of the earlier osteogenic marker ALP and the late osteogenic markers OCN protein were obviously enhanced in fibroblasts + 1 mg/ml UHMWPE particles group compared to control group (fibroblasts only). However, the expression of the ALP and OCN protein gradually decreased as the ratio of macrophage supernatants vs. fibroblast completed media increased (Fig. 4a). ELISA results confirmed that the expressions of ALP and OCN were slightly reduced in fibroblasts + 1/16 and 1/8 macrophage supernatants group compared with fibroblasts + 1 mg/ml UHMWPE particles group (*p* > 0.05, Fig. 4b); however, a significant decrease was found while 1/4, 1/2, and 1/1 macrophage supernatants were added compared with fibroblasts + 1 mg/ml UHMWPE particles group (*p* < 0.05).

## Discussion

Periprosthetic osteolysis, followed by implant loosening, is induced by UHMWPE wear particles and results in the failure of THA and the necessity of total joint revision procedures [20]. Related studies reported that approximately 10–50% of all revision THA can be attributed to periprosthetic osteolysis [21]. Wear debris, generated by articulating motion at the bearing surfaces, induces progressive osteolysis by increasing bone resorption and suppressing bone formation [22, 23]. Therefore, it is urgently essential to address the problem for the patients with end-stage osteoarthritis, avascular necrosis of the hip, or femoral neck fractures in THA [24]. The purpose of this study is to assess the effects of macrophages on the osteogenic capability of fibroblasts treated with UHMWPE wear particles. Our results confirmed that macrophages participate in the periprosthetic osteolysis process by reducing the osteogenic capability of fibroblasts treated with UHMWPE wear particles, and involved in the implant loosening. Currently, a variety of cells have been identified in the periprosthetic interfacial membrane (bone-implant interface), such as histiocytes, fibroblasts, endothelial cells, lymphocytes, osteoblasts, osteoclasts, and macrophages, while synovial-like membrane mainly consists of macrophages and fibroblasts [25, 26].

Our previous study demonstrated that fibroblasts stimulated by UHMWPE wear particles (1 mg/ml) have the capacity of forming osteoblast to reduce osteolysis [15]. It provided favorable evidence for clinical use of UHMWPE as prosthesis in THA as reported by other literature [27, 28]. It is well known that the macrophage response to UHMWPE wear debris plays a critical role in the long-term periprosthetic osteolysis [29]. Prosthetic particles, produced by wear at the articulating surface of prostheses are phagocytosed by macrophages [30]. This study was designed to investigate how macrophages participate in the processes of fibroblasts to osteoblast by stimulating UHMWPE wear particles.

Osteoblasts play a critical role in periprosthetic osteolysis, as normal bone metabolism commonly relies on the balance between bone formation and degradation, and either increased bone resorption or decreased bone formation can lead to the loss of bone stock [31]. Wear particles have been presented to exert a negative effect on osteoblasts during the process of osteolysis. In our work, fibroblasts were successfully separated from the hip synovial membrane during THA or revision procedures, and UHMWPE particles with desired sizes (average diameter 6.54 μm ± 4.43 μm) and various shapes were prepared through micro-grinding procedures. MTT results indicated that the survival rate of macrophages was significantly higher when it was treated with 0.1 mg/ml UHMWPE particles. Findings of the present study are consistent with Zaveri et al. [32] who reported that the optimum proliferation effect of fibroblasts for co-culture was stimulated by 0.1 mg/ml UHMWPE particles. A study by an American reported that the RAW264.7 cells with a density of 1 × 10^5^/mL were most sensitive to the 0.1 mg/mL concentration of UHMWPE [33]. Macrophages are responsible for mediating foreign body immune reaction to wear particles from joint replacements [26]. Many studies reported that UHMWPE wear particles induced macrophage infiltration and inflammation leading to periprosthetic osteolysis through secreting inflammatory cytokines [29, 34]. According to the proportion of macrophage supernatants in fibroblast complete media, the co-culture system was divided into seven groups, besides the fibroblasts only group and fibroblasts + 1 mg/ml UHMWPE particles group. MTT results further indicated that fibroblasts treated with 1 mg/ml UHMWPE particles containing 1/16 of the corresponding macrophage supernatants had the highest cell viability among the macrophage supernatant treatment groups. We tried to mimic the effective joint space environment by culture of the fibroblasts with macrophage supernatants and UHMWPE wear particles. To date, methods for the treatment of wear debris-induced osteolysis have focused on the inhibition of inflammation and target osteoclasts [35]. ALP is a traditional marker of osteoblast differentiation and is related to the production of a mineralized osteoblast [36]. The Western blot and ELISA analysis were combined to explore the osteogenic ability of fibroblasts in the protein level, with mainly focusing on the ALP and OCN. Our results suggested that the osteogenic ability of fibroblasts treated with 1 mg/ml UHMWPE particles was reduced by adding macrophage supernatants to fibroblast complete media, just as the alizarin red staining showed. Wang et al. suggested that a lower expression of ALP and OCN in osteoprogenitor cells stimulated with Ti particles [37].

However, there are several limitations to our study. First is to mimic the effective joint space environment scenario. However, it has been demonstrated that these particles induced osteolysis around the prosthetic implant by mechanisms similar to polyethylene particles. An animal model study with continuous particle generation would provide a better conclusion in the future.

## Conclusions

Thus, based on the data presented, we speculated that macrophages participate in the periprosthetic osteolysis process by reducing the osteogenic capability of fibroblasts treated with UHMWPE wear particles and involved in the implant loosening. This work strengthened our understanding about the complex bone-implant microenvironment in total hip arthroplasty.

## Abbreviations

ALP: Alkaline phosphatase
BMP 2: Bone morphogenetic protein-2
DMSO: Dimethyl sulfoxide
FBS: Fetal bovine serum
HRP: Horseradish peroxidase
MC3T3: Mouse clonal osteoblast-like cells
OCN: Osteocalcin
PMSF: Phenylmethylsulphonyl fluoride
PVDF: Polyvinylidene difluoride
THA: Total hip arthroplasty
UHMWPE: Ultrahigh molecular weight polyethylene
